# AICAR Enhances the Phagocytic Ability of Macrophages towards Apoptotic Cells through P38 Mitogen Activated Protein Kinase Activation Independent of AMP-Activated Protein Kinase

**DOI:** 10.1371/journal.pone.0127885

**Published:** 2015-05-28

**Authors:** Hui Quan, Joung-Min Kim, Hyun-Jung Lee, Seong-Heon Lee, Jeong-Il Choi, Hong-Beom Bae

**Affiliations:** 1 Departments of Anesthesiology and Pain Medicine, Chonnam National University Medical School, Gwangju, Republic of Korea; 2 Research Institute of Medical Sciences, Chonnam National University, Gwangju, Republic of Korea; Institute of Biochemistry and Biotechnology, TAIWAN

## Abstract

Recent studies have suggested that 5-aminoimidazole-4-carboxamide-1-β-D-ribofuranoside (AICAR) increases macrophage phagocytosis through adenosine monophosphate-activated protein kinase (AMPK). However, little information is available on the effects of AICAR on the clearance of apoptotic cells by macrophages, known as efferocytosis, which is essential in maintaining tissue homeostasis and resolving inflammation. AICAR increased p38 MAPK activation and the phagocytosis of apoptotic cells by macrophages, which were inhibited by the p38 MAPK inhibitor, SB203580, the TGF-beta-activated kinase 1 (TAK1) inhibitor, (5Z)-7-oxozeaenol, and siRNA-mediated knock-down of p38α. AICAR increased phosphorylation of Akt, but the inhibition of PI3K/Akt activity using LY294002 did not affect the AICAR-induced changes in efferocytosis in macrophages. CGS15943, a non-selective adenosine receptor antagonist, did not affect AICAR-induced changes in efferocytosis, but dipyridamole, an adenosine transporter inhibitor, diminished the AICAR-mediated increases in efferocytosis. AICAR-induced p38 MAPK phosphorylation was not inhibited by the AMPK inhibitor, compound C, or siRNA-mediated knock-down of AMPKα1. Inhibition of AMPK using compound C or 5’-iodotubercidin did not completely block AICAR-mediated increases in efferocytosis. Furthermore, AICAR also increased the removal of apoptotic neutrophils or thymocytes in mouse lungs. These results reveal a novel mechanism by which AICAR increases macrophage-mediated phagocytosis of apoptotic cells and suggest that AICAR may be used to treat efferocytosis-related inflammatory conditions.

## Introduction

Engulfment and clearance of apoptotic cells by phagocytes, a process known as efferocytosis, is essential to maintain tissue homeostasis and resolve inflammatory conditions [1, 2]. In contrast to the uptake of pathogens, macrophages that engulf apoptotic cells produce anti-inflammatory cytokines, such as transforming growth factor β (TGF-β) and interleukin 10 (IL-10), which dampen inflammation and inhibit inflammatory mediator production [1–3]. If dying cells are not cleared effectively, they result in secondary necrotic cells followed by leakage of harmful intracellular contents into the local environment, which impedes the resolution of inflammation and wound healing. Recent studies have shown that ineffective efferocytosis is associated with acute lung injury, COPD and cystic fibrosis [4–7].

Although the engagement of phagocytic receptors with apoptotic cells increases Rac1 activity and decreases RhoA activity, which are involved in cytoskeletal reorganization during the engulfment of apoptotic cells [1, 8, 9], the mechanisms involved in intracellular signaling events during efferocytosis are not well-defined. Studies have reported that activation of p38 mitogen activated protein kinase (MAPK) induces actin cytoskeletal reorganization, which is involved in cell migration and phagocytosis in various cell populations [10–14]. For example, the retinoic acid-induced increase in p38 MAPK activity is involved in cytoskeletal remodeling and glucose uptake in skeletal muscle cells [13]. P38 MAPK was also involved in the phagocytosis of apoptotic spermatogenic cells via increases in GTP-bound Rac1 [14].

The compound 5-aminoimidazole-4-carboxamide-1-β-D-ribofuranoside (AICAR) is a cell-permeable adenosine analog that is taken up by cells through an adenosine transporter. This compound is phosphorylated rapidly by adenosine kinase to form 5-aminoimidazole-4-carboxamide ribotide monophosphate (ZMP), which increases adenosine monophosphate-activated protein kinase (AMPK) activity by mimicking AMP [15, 16]. Previous studies have suggested that AMPK activation using AICAR reduced Toll-like receptor (TLR) 2/4 activation-induced inflammatory responses *in vitro* and *in vivo* [17–20]. However, AICAR is thought to increase the expression of peroxisome proliferator-activated receptor α-responsive genes in hepatocytes and to decrease TLR4-induced TNF-α production, and iNOS and COX-2 gene transcription in macrophages through an AMPK-independent mechanism [21–23]. These results suggest that some biological actions of AICAR do not require modulation of AMPK activity, although AICAR is commonly used as a pharmacological activator of AMPK.

In this study, we investigated the effects of AICAR on the ability of macrophages to eliminate apoptotic cells. We found that AICAR increased p38 MAPK activities, independently of AMPK in macrophages, which was associated with increased efferocytosis. These results provide novel insights into the role of AICAR in phagocytosis of apoptotic cells in macrophages.

## Materials and Methods

### Ethics statement

All animal experiments were approved by the Animal Care and Ethics Committee of Chonnam National University Medical School and followed the guidelines by the U.S. National Institutes of Health Guide for the Care and Use of Laboratory Animals.

### Mice

Male BALB/c mice (20~25 g, 8–10 weeks) were purchased from Samtako Science (Daejeon, Korea). The mice were kept on a 12-h light/dark cycle with free access to food and water. All experiments were conducted in accordance with the institutional review board-approved protocols.

### Reagents

AICAR was purchased from Enzo Life Sciences (Plymouth Meeting, PA, USA). RPMI 1640 with L-glutamine, DMEM, penicillin-streptomycin and fetal bovine serum (FBS) were obtained from GIBCO (Gaithersburg, MD, USA). Bicinchoninic acid (BCA) protein assay reagent was obtained from Pierce (Rockford, IL, USA). Antibodies specific for p-AMPK (Thr^172^), p-p38 (Thr^180^/Tyr^182^), p-MKK3/6 (Ser^189^/Ser^207^), AMPK and p38, and compound C were purchased from Cell Signaling Technologies (Beverly, MA, USA). CGS15943, 5’-iodotubercidin, (5z)-7-oxozeaenol and dipyridamole were from Sigma-Aldrich (St Louis, MO, USA). SB203580 was obtained from Calbiochem (La Jolla, CA, USA). Control siRNA and siRNA to the AMPKα1 and p38α were purchased from Santa Cruz Biotechnology (Santa Cruz, CA, USA). Custom antibody mixtures and negative selection columns for neutrophil isolation were purchased from Stem cell Technologies (Vancouver, British Columbia, Canada).

### Cell preparation

Mouse bone marrow neutrophils were purified using a customized negative selection kit (Stem Cell Technologies) as previously described [5, 24]. Neutrophil purity, as determined by Wright-Giemsa-stained cytospin preparations, was consistently greater than 97%. Neutrophil viability under experimental conditions was determined using trypan blue staining and was consistently > 97%. Peritoneal macrophages were obtained from 8–10 week old mice using Brewer thioglycollate as previously described [25]. Briefly, cells were collected 4 days after intraperitoneal injection of Brewer thioglycollate and were cultured in 12-well plates (1 x 10^6^ cells/well) in RPMI 1640 medium containing 5% FBS, 100 units/ml penicillin, and 100 μg/ml streptomycin at 37°C. After 1 h, non-adherent cells were removed by washing with culture medium. The murine macrophage RAW264.7 was obtained from the Korea Cell Line Bank (Seoul, Korea). Cells were cultured in DMEM media with 10% FBS, 100 units/ml penicillin, and 100 μg/ml streptomycin at 37°C. The thymi were removed from mice and transferred to a culture dish with RPMI 1640 medium containing 5% FBS, 100 units/ml penicillin, and 100 μg/ml streptomycin and were minced into about 3–4 mm pieces with sterile scissors. Tissue pieces were transferred to 40 μm cell strainer and followed by gentle grinding of the tissue across the mesh with plunger of 3 ml syringe. Thymocytes suspensions were obtained by passing culture media and cells through cell strainer.

### Western blot analysis

Western blot analysis was performed as previously described [24, 26]. Briefly, equal amounts of protein were separated by 8–10% SDS-PAGE, and electrotransferred onto polyvinylidene difluoride (PVDF) membranes. To determine the levels of total and phosphorylated proteins, membranes were probed with specific primary antibodies followed by detection with HRP-conjugated goat anti-rabbit IgG secondary antibodies. Bands were detected using enhanced chemiluminescence (ECL) western blotting detection reagents (Millipore, Billerica, MA, USA), and imaged with LAS-3000 (Life Science Systems, Fujifilm Global). Densitometry was performed using a Multi gauge V3.0 chemiluminescence system and analysis software (Life Science Systems, Fujifilm Global) to quantify the ratio between phosphorylated and total proteins.

### In vitro efferocytosis assay

Phagocytosis of apoptotic neutrophils was determined as previously described [25]. Briefly, apoptotic neutrophils were obtained by incubation in RPMI 1640 with 1% FBS at 43°C for 1 h followed by culture for an additional 2.5 hours at 37°C. Using this method, > 70% of the neutrophils were apoptotic as determined by annexin V and propidium iodide staining. 2.5 × 10^6^ apoptotic neutrophils were added to 2.5 × 10^5^ macrophages cultured on coverslips in RPMI 1640 medium with 0.5% FBS, at 37°C for 2 h. The coverslips were vigorously washed 3 times with ice-cold PBS and stained with HEMA3. Phagocytosis was evaluated by an observer blinded to experimental conditions through counting 200–300 macrophages/slide. The phagocytic index was represented as the percentage of macrophages containing at least 1 ingested neutrophil.

Apoptotic thymocytes were obtained by treatment with dexamethasone, as previously described [5, 27]. Briefly, mouse thymocytes were labeled using a PKH26 Red Fluorescent Dye Linker kit (Sigma-Aldrich), according to the manufacturer’s instructions, and then the thymocytes were resuspended in RPMI 1640 medium with 10% FBS and 1 *μ*M dexamethasone at a concentration of 6 × 10^6^ cells/ml and incubated at 37°C in 5% CO_2_ for 16 h. Using this method, > 90% of the thymocytes were early or late apoptotic as determined by annexin V and propidium iodide staining. Phagocytosis of apoptotic thymocytes was determined by adding two fold excess of apoptotic thymocytes to 7 × 10^5^ macrophages or 3 × 10^5^ RAW 264.7 cells in RPMI-medium or DMEM-medium with 0.5% FBS, respectively, at 37°C for 90 min. Noningested cells were removed by washing three times with ice-cold PBS. Macrophages were detached from the wells using 0.25% trypsin/EDTA in PBS, centrifuged and the cell pellet was re-suspended in PBS containing 1% albumin, FITC-conjugated CD11b (macrophage marker) antibody and APC-conjugated CD90.2 (thymocyte marker) antibody. Flow cytometry was performed. The phagocytic index was calculated as the ratio of FITC^+^PKH26^+^APC^-^ cells to FITC^+^ cells gated. Engulfed thymocytes are not accessible to the APC-conjugated CD90.2 antibody. Therefore, FITC^+^PKH26^+^APC^-^ cells are macrophages that have engulfed PKH-labeled thymocytes, whereas the APC^+^PKH^+^FITC^+^ cells were macrophages, which thymocytes are adherent to but are not engulfed.

### In vivo efferocytosis assay

To determine phagocytosis of apoptotic neutrophils or apoptotic thymocytes *in vivo* condition, mice were anesthetized with sevoflurane, and 5 × 10^6^ apoptotic neutrophils or PKH26 labeled apoptotic thymocytes in 50 μl PBS were administered to mice lung via tracheotomy 4 h after intraperitoneal injection of AICAR (500 mg/kg) or vehicle in 0.2 ml of PBS as previously described [5, 25]. To measure phagocytosis of apoptotic neutrophils, alveolar cells were collected 2 h after administration of apoptotic neutrophils by lavaging the lungs three times with 1 ml of iced PBS with 5 mM EDTA. Cytospin slides were prepared using 100 μl of the bronchoalveolar lavage (BAL) fluid and stained with HEMA3, and phagocytosis index was determined. To measure phagocytosis of apoptotic thymocytes, bronchoalveolar lavage fluids were collected 90 min after intratracheal administration of apoptotic thymocytes and stained with FITC-conjugated CD11b antibody and APC-conjugated CD90.2 antibody. Phagocytic index was determined using flow cytometry.

### siRNA knock down of AMPKα1 and p38α MAPK

RAW 264.7 cells (3 × 10^5^/well) in 12 well plates were incubated in Lipofectin (Gibco-BRL, Rockville, MD, USA) with 200 nM control siRNA or siRNA specific to AMPKα1 and p38α MAPK for 48 h. Cells were treated as described in the figure legends and then followed by western blot analysis or efferocytosis assay.

### ELISA

IL-10 levels in BAL fluids were quantified by ELISA kits obtained from R&D Systems (Minneapolis, MN, USA) according to the manufacturer’s instructions.

### Statistical analysis

Data from the experiments were expressed as means ± standard deviation (SD). Statistical significances were determined using the Student’s *t* test for comparisons of two groups. Multi-group comparisons were performed using one-way ANOVA with Turkey’s post hoc test (SPSS software version 21.0). A value of *P* < 0.05 was considered significant.

## Results

### AICAR increases the phagocytic ability of macrophages toward apoptotic cells through activation of p38 MAPK in macrophages

**Fig 1:**
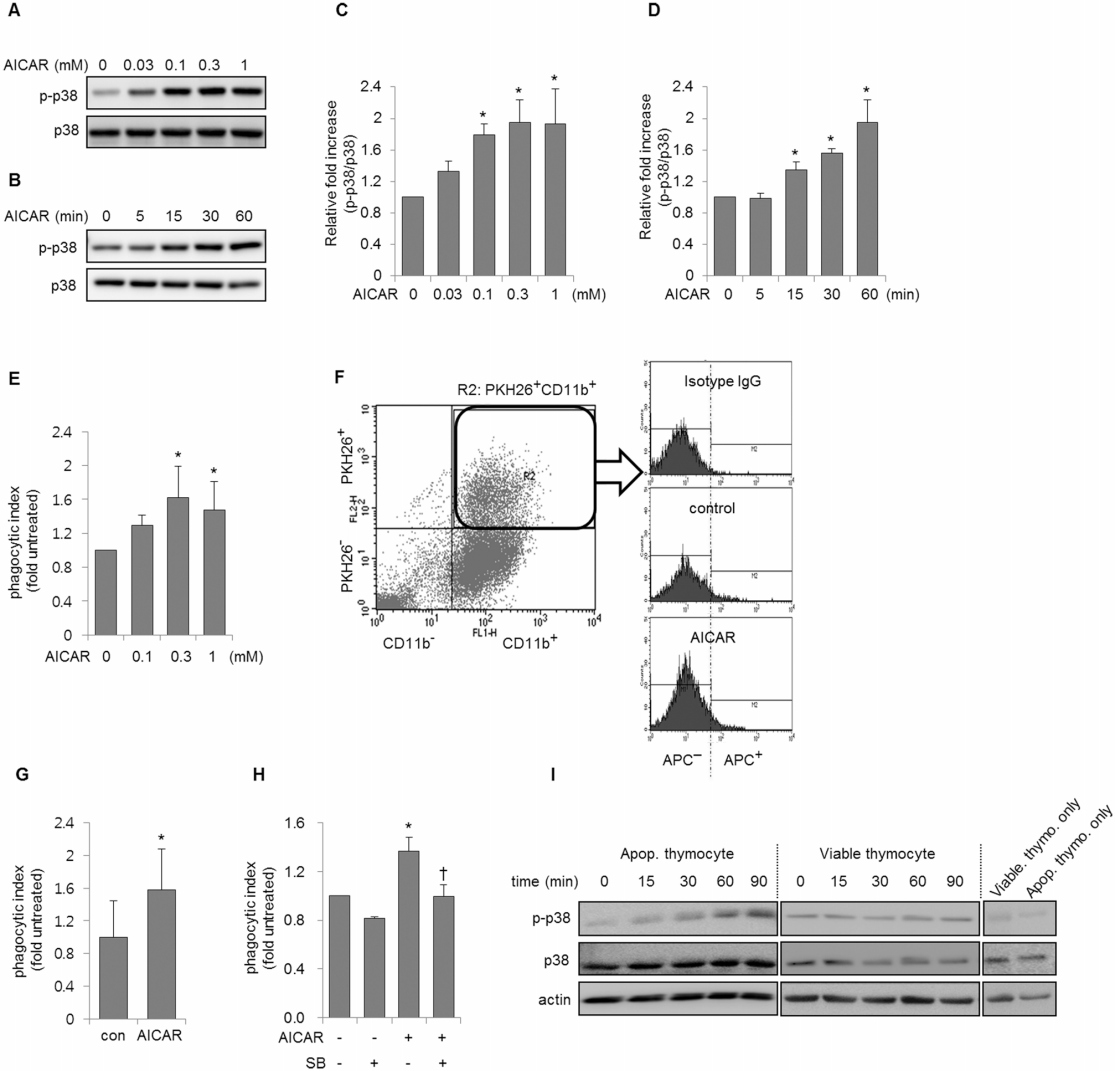
Effects of 5-aminoimidazole-4-carboxamide-1-β-D-ribofuranoside (AICAR) on phagocytosis of apoptotic cells by macrophages. **(A-D)** Peritoneal macrophages were cultured **(A)** with AICAR at the indicated doses for 1 h or **(B)** with AICAR (0.3 mM) for the indicated times. Cell lysates were subjected to Western blotting using antibodies directed against phosphor (Thr^180^/tyr^182^)—and total p38 mitogen activated protein kinases (MAPKs). **(C, D)** The density ratio of phosphorylated to total p38 MAPK was calculated using data from four independent experiments. Each bar represents the mean ± SD. *P < 0.05 compared to the control. **(E)** Macrophages were cultured with AICAR at the indicated doses for 1 h and then incubated with apoptotic thymocytes (two-fold excess over the cell number) for 90 min. The phagocytic index was determined using flow cytometry as described in the Materials and Methods. Each bar represents the mean ± SD (n = 4). *P < 0.05 compared to the control. **(F)** Representative flow cytometry histograms from efferocytosis assays of apoptotic thymocytes are shown. **(G)** Macrophages were cultured with AICAR (0 or 0.3 mM) for 1 h and then incubated with apoptotic neutrophils (10-fold excess over the cell number) for 2 h. The phagocytic index was determined as described in the Materials and Methods. Means ± SD were obtained from four independent experiments. *P < 0.05 compared with control (untreated cells). **(H)** Peritoneal macrophages were incubated with AICAR (0 or 0.3 mM) 1 h after treatment with SB203580 (SB: p38 MAPK inhibitor, 0 or 10 μM), and then cultured with apoptotic thymocytes for an additional 90 min. *P < 0.05 compared with untreated cells. ^†^ *P* < 0.05 compared with cells treated with AICAR only. Each bar represents the mean ± SD (n = 4). **(I)** Macrophages were cultured with apoptotic or viable thymocytes (two-fold excess over the cell number) for the indicated times followed by Western blot analysis. Representative gels show p38 MAPK phosphorylation of macrophages cultured with apoptotic or viable thymocytes. The status of p38 MAPK phosphorylation in viable thymocytes (Viable thymo. Only) or apoptotic thymocytes (Apop. Thymo. Only) was measured. Two additional experiments gave similar results.

**Fig 2:**
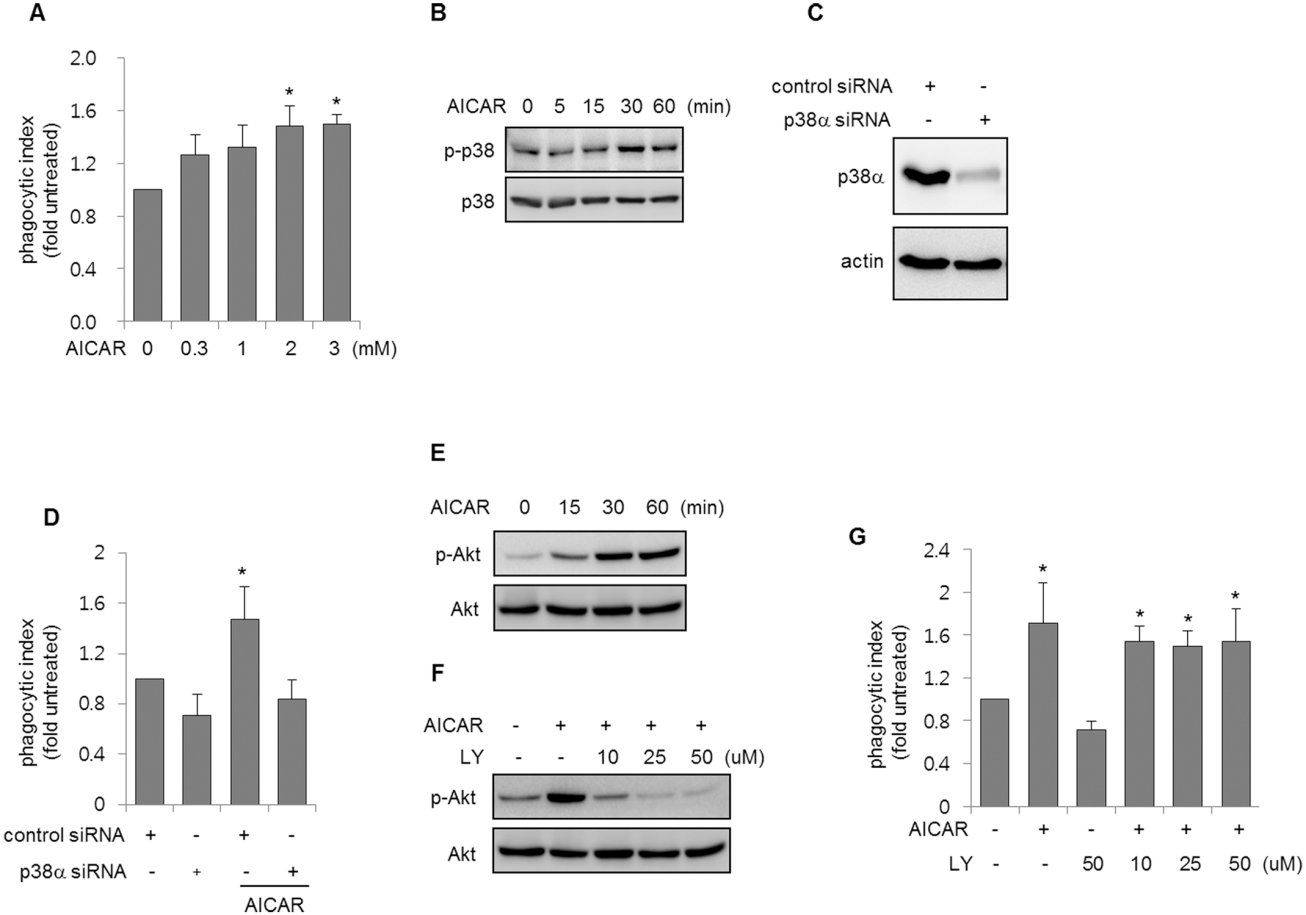
Inhibition of p38 MAPK activity suppresses AICAR-induced efferocytosis. **(A)** RAW 264.7 cells were cultured with AICAR at the indicated doses for 1 h and then incubated with apoptotic thymocytes for 90 min followed by flow cytometry. Each bar represents the mean ± SD (n = 4). **(B)** RAW 264.7 cells were cultured with AICAR (2 mM) for the indicated times. Cell lysates were subjected to Western blot analysis using specific antibodies. **(C)** Representative Western blots show the levels of p38α or actin in RAW 264.7 cells treated with control siRNA or siRNA specific to p38α. **(D)** RAW 264.7 cells treated with control siRNA or siRNA specific to p38α were incubated with AICAR (0 or 2 mM) for 1 h and then cultured with apoptotic thymocytes for an additional 90 min, followed by flow cytometry. *P < 0.05 compared with the control. ^†^ *P* < 0.05 compared with control cells treated with AICAR. Each bar represents the mean ± SD (n = 4). **(E)** Peritoneal macrophages cultured with AICAR (0.3 mM) for the indicated times and subjected to Western blot analysis. **(F, G)** Macrophages were cultured with LY294002, a PI3K inhibitor, at the indicated concentrations for 30 min before exposure to AICAR (0 or 0.3 mM) for 1 h, followed by **(F)** Western blot analysis or **(G)** culturing with apoptotic thymocytes for an additional 90 min. The phagocytic indexes were measured by flow cytometry. Values represent the mean ± SD from four independent experiments. *P < 0.05 compared with untreated cells.

We explored whether AICAR increased p38 MAPK activity in macrophages. AICAR induced a dose- and time-dependent increase in p38 MAPK phosphorylation in mouse peritoneal macrophages (Fig 1A–1D). AICAR also enhanced the phagocytosis of apoptotic thymocytes and neutrophils, in parallel with the increased p38 MAPK activity (Fig 1E–1G). The presence of SB203580, a specific p38 MAPK inhibitor, in macrophage cultures before adding AICAR reduced the AICAR-mediated increase in efferocytosis (Fig 1H). These results are consistent with studies suggesting that p38 MAPK is associated with cytoskeletal reorganization, which is involved in general phagocytic processes [10, 14, 28–30]. As shown in Fig 1I, p38 MAPK phosphorylation increased in cultures of macrophages with apoptotic thymocytes, but co-culture of macrophages with viable thymocytes did not affect p38 MAPK phosphorylation. These results suggest that p38 MAPK is involved in macrophage efferocytosis. Additional experiments showed that AICAR increased the efferocytosis of apoptotic thymocytes and phosphorylation of p38 MAPK in RAW 264.7 cells (Fig 2A and 2B). Knock-down of p38α in RAW 264.7 cells also abolished the effect of AICAR on efferocytosis (Fig 2C and 2D). Previous studies suggested that AICAR increases PI3k/Akt activity in macrophages, and PI3K/Akt may be involved in the phagocytosis of apoptotic cells by macrophages [31–33]. We examined whether AICAR could increase PI3K/Akt activity in peritoneal macrophages. As shown in Fig 2E and 2F, AICAR rapidly increased the phosphorylation of Akt, while the PI3K inhibitor, LY294002, prevented the phosphorylation of Akt by AICAR in macrophages, suggesting that AICAR-induced Akt phosphorylation is associated with PI3K activation in macrophages. However, the inhibition of PI3K/Akt activity by LY294002 did not block AICAR-mediated increases in efferocytosis in macrophages (Fig 2G). These results indicate that PI3k/Akt activation is not an essential component of AICAR-induced efferocytosis.

### Intracellular AICAR uptake is required to increase efferocytosis in peritoneal macrophages

**Fig 3:**
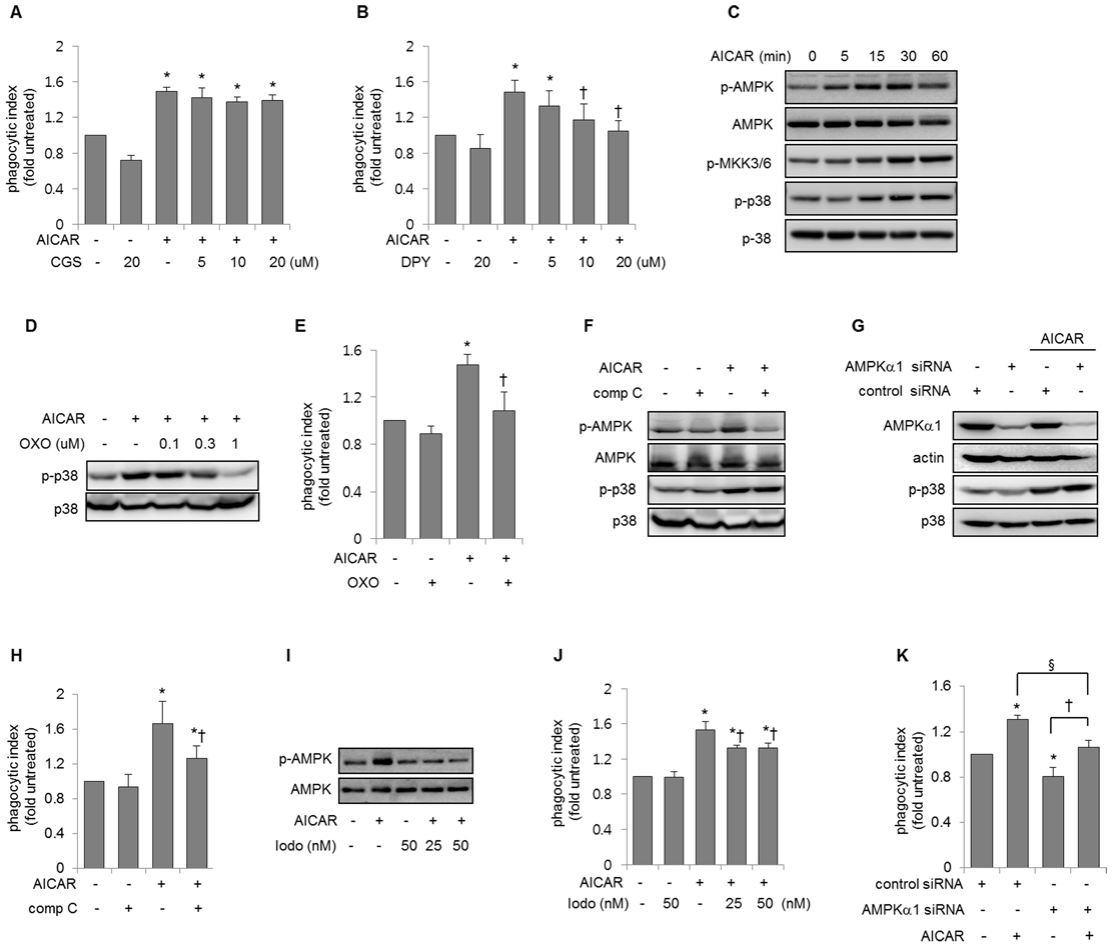
AICAR-induced activation of p38 MAPK pathways does not require adenosine monophosphate-activated protein kinase (AMPK) activation. **(A)** Peritoneal macrophages were cultured with CGS15943 (CGS: non-selective adenosine receptor antagonist) at the indicated doses for 15 min before exposure to AICAR (0 or 0.3 mM), followed by culture with apoptotic thymocytes for an additional 90 min. Means ± SD are shown (n = 4). *P < 0.05 compared with untreated cells **(B)** Peritoneal macrophages were cultured with dipyridamole (DPY; an adenosine transporter inhibitor) at the indicated doses for 30 min before exposure to AICAR (0 or 0.3 mM) for 1 h, after which cells were incubated with apoptotic thymocytes for an additional 90 min. The phagocytic index was determined by flow cytometry. Means ± SD are shown (n = 4). *P < 0.05 compared with untreated cells. ^†^ *P* < 0.05 compared with cells treated with AICAR only. **(C)** Peritoneal macrophages were cultured with AICAR for the indicated times, and cell lysates were subjected to Western blotting using antibodies specific to phospho-AMPK (Thr^172^), phospho-p38 (Thr^180^/tyr^182^), phospho-MKK3/6 (Ser^189/207^), AMPK, p38 MAPK or actin. **(D)** Cells were cultured with (5Z)-7-oxozeaenol (Oxo; a TAK 1 inhibitor) at the indicated concentrations for 1 h before exposure to AICAR (0 or 0.3 mM) for 1 h. Cell lysates were analyzed by Western blot, and representative Western blots are shown. **(E)** Peritoneal macrophages were incubated with AICAR (0 or 0.3 mM) 1 h after treatment with Oxo (0 or 1 μM), and then cultured with apoptotic thymocytes for an additional 90 min. Each bar represents the mean ± SD (n = 4). *P < 0.05 compared with untreated cells. ^†^ *P* < 0.05 compared with cells treated with AICAR only. **(F)** Cells were cultured with compound C (comp C, 0 or 10 μM) for 2 h before exposure to AICAR (0 or 0.3 mM) for 1 h. Cell lysates were analyzed by Western blot. **(G)** Representative Western blots show the effect of AICAR on phosphorylation of p38 in RAW 264.7 cells treated with nonspecific control siRNA or siRNA specific to AMPKα1. **(H)** Peritoneal macrophages were cultured with compound C (comp C, 0 or 10 μM) for 2 h before adding AICAR (0 or 0.3 mM) for 1 h and then incubated with apoptotic thymocytes for 90 min. The phagocytic index was determined using flow cytometry. Means ± SD are shown (n = 5). **(I, J)** Peritoneal macrophages were treated with 5’-iodotubericidin (Iodo: an adenosine kinase inhibitor) at the indicated doses for 1 h followed by the addition of AICAR (0 or 0.3 mM) for 1 h, after which **(I)** cell lysates were subjected to Western blotting or **(J)** incubated with apoptotic thymocytes for an additional 90 min, followed by flow cytometry to determine the phagocytic index. Means ± SD are shown (n = 4). *P < 0.05 compared with untreated cells. ^†^ *P* < 0.05 compared with cells treated with AICAR only. **(K)** RAW 264.7 cells treated with control siRNA or siRNA specific to AMPKα1 were incubated with AICAR (0 or 2 mM) for 1 h and then cultured with apoptotic thymocytes for an additional 90 min followed by flow cytometry. *P < 0.05 compared with the control. ^†^ *P* < 0.05, ^§^ *P* < 0.05.

Previous studies have suggested that AICAR competes with released adenosine for uptake by the adenosine transporter, which results in increased adenosine concentrations in the culture medium and subsequent activation of adenosine receptors [15, 34]. To address this issue, we explored whether inhibition of adenosine receptors affects the AICAR-induced changes in macrophage efferocytosis. As shown in Fig 3A, CGS15943, a non-selective adenosine receptor antagonist, did not inhibit the effects of AICAR on macrophage efferocytosis. AICAR enters cells via an adenosine transporter and is phosphorylated by adenosine kinase [15, 16]. Therefore, we investigated whether the effect of AICAR on efferocytosis depends on its intracellular uptake. As shown in Fig 3B, dipyridamole, an adenosine transporter inhibitor, diminished AICAR-induced increases in efferocytosis (Fig 3B).

### AICAR increases activation of p38 MAPK pathways independently of AMPK in macrophages

Studies have demonstrated that AMPK activation enhanced the phagocytosis of apoptotic cells and might also interact with stress-signaling pathways such as p38 MAPK that can involve in engulfment of apoptotic cells [14, 25, 28, 30]. We explored whether the AICAR-induced increase in the p38 MAPK activity in macrophages is dependent on AMPK. As shown in Fig 3C, AICAR time-dependently increased the phosphorylation of AMPK, p38 MAPK, and MAPK kinase 3/6 (MKK3/6), a kinase upstream of p38 MAPK. In addition, the inhibition of TGF-β-activated kinase 1 (TAK1) (an upstream signal of MKK3/6 and p38 MAPK) activity using (5Z)-7-oxozeaenol suppressed AICAR-induced p38 MAPK phosphorylation in a dose-dependent manner (Fig 3D). The AICAR-induced increase in efferocytosis was decreased by inhibiting TAK1 activation using (5Z)-7-oxozeaenol (Fig 3E). However, the presence of compound C, an AMPK inhibitor, in the macrophage cultures resulted in decreased phosphorylation of AMPK by AICAR, but not p38 MAPK (Fig 3F). AICAR also increased the phosphorylation of p38 MAPK in AMPKα1 knock-down RAW 264.7 cells (Fig 3G), suggesting that p38 MAPK phosphorylation by AICAR is independent of AMPK activity. The presence of compound C in macrophage cultures before adding AICAR diminished the AICAR-mediated increase in efferocytosis, but not completely (Fig 3H). In addition, 5’-iodotubercidin, an adenosine kinase inhibitor, inhibited the conversion of AICAR to ZMP, a direct activator of AMPK, according to the decreased phosphorylation of AMPK (Fig 3I). Similar to the results obtained using compound C, 5’-iodotubercidin significantly, but only partially, suppressed the AICAR-induced increase in efferocytosis in peritoneal macrophages (Fig 3J). AMPKα1 siRNA decreased the basal phagocytic index as well as the AICAR-induced increase in the phagocytic index (Fig 3K).

### AICAR increases the clearance of apoptotic cells in vivo

**Fig 4:**
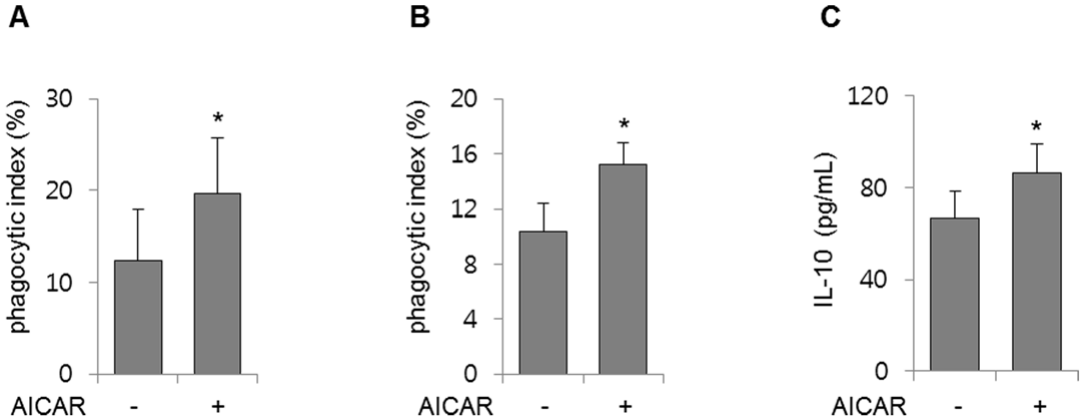
Effects of AICAR on the removal of apoptotic cells *in vivo*. Mice were injected intraperitoneally with AICAR (0 or 500 mg/kg) 4 h before intratracheal administration of apoptotic neutrophils or thymocytes. **(A)** Bronchoalveolar (BAL) fluids were collected 2 h after administration of apoptotic neutrophils. Cytospin slides were prepared from BAL fluid and stained with HEMA3, and the phagocytosis index was determined as described in the Materials and Methods. **(B)** BAL fluids were collected 90 min after administration of PKH26-labelled apoptotic thymocytes. Samples were stained with FITC-conjugated CD11b antibody and APC-conjugated CD 90.2 antibody. The phagocytic index was calculated as described in the Materials and Methods. (C) BAL fluids were collected 4 h after administration of apoptotic thymocytes and the IL-10 levels in the BAL fluids were determined using an enzyme-linked immunosorbent assay. Values represent the mean ± SD from five mice per group. *P < 0.05 compared with the control group.

Given that AICAR increased macrophage efferocytosis *in vitro*, we determined whether AICAR increases the phagocytic ability of macrophages toward apoptotic cells *in vivo*. Mice were subjected to intraperitoneal AICAR prior to intratracheal administration of apoptotic neutrophils or thymocytes. As shown in Fig 4A, elimination of apoptotic neutrophils increased significantly in mouse lungs treated with AICAR compared with control mice. To further characterize the effect of AICAR on efferocytosis *in vivo*, mice were injected intratracheally with PKH26-labeled apoptotic thymocytes after intraperitoneal administration of AICAR. As shown in Fig 4B, removal of apoptotic thymocytes increased significantly in mice lungs treated with AICAR compared with control mice. The IL-10 levels in BAL fluids 4 h after intratracheal injection of apoptotic thymocytes increased in mouse lungs treated with AICAR compared with control mice (Fig 4C). These findings indicate that AICAR increases the release of the anti-inflammatory cytokine IL-10, which is associated with AICAR induced increases in efferocytosis.

## Discussion

We found that AICAR increased the activity of p38 MAPK in macrophages and enhanced phagocytosis of apoptotic cells *in vitro* and *in vivo*. AICAR-induced p38 MAPK activation did not require AMPK activation in macrophages, since neither compound C nor siRNA-induced knock-down of AMPKα1 inhibited p38 MAPK phosphorylation by AICAR. Phagocytosis of apoptotic cells by macrophages was also suppressed by inhibiting p38 MAPK activation. In cultured macrophages, AICAR increased the activity of AMPK and the uptake of apoptotic cells. These results concur with observations that AMPK activation enhances efferocytosis [35]. However, the AICAR-induced increase in efferocytosis was not blocked completely by the AMPK inhibitor compound C, siRNA-mediated knockdown of AMPKα1, or the adenosine kinase inhibitor 5’-iodotubercidin, which inhibits the conversion of AICAR to ZMP; this mimicked the effect of AMP on allosteric activation of AMPK and the induction of AMPK phosphorylation by AMPK kinase [15, 34]. These results demonstrated that p38 MAPK activation is directly involved, while AMPK activation was only indirectly involved, in the AICAR-induced increase in macrophage efferocytosis.

Previous studies have demonstrated that AMPK promotes p38 MAPK activation in various cell populations [13, 36–38]. For example, overexpression of the constitutively active form of mutant AMPK increased p38 MAPK phosphorylation in rat liver cells [36]. But, several studies have shown that AICAR has biological activities independent of AMPK [21–23]. AICAR also increased the phosphorylation of p38 MAPK, but it was not involve in the suppressive effect of AICAR on GM-CSF-induced macrophage proliferation via AMPK activation [39]. In the present experiments, AICAR increased phosphorylation of p38 MAPK and MKK 3/6 in parallel with AMPK phosphorylation in cultured macrophages. However, the effect of AICAR on p38 MAPK was not blocked by compound C. Similarly, AICAR-induced phosphorylation of p38 MAPK was not prevented by siRNA-mediated knock-down of AMPKα1, which is the predominant AMPK catalytic subunit in peritoneal macrophages and RAW 264.7 cells because AMPKα2 is deficient or present at low concentrations [22, 25, 35]. These results indicate that AICAR-induced p38 MAPK activation does not require AMPK activity in macrophages, although AMPK can regulate p38 MAPK activation.

In these experiments, AICAR-induced increase in efferocytosis was suppressed by inhibiting p38 MAPK using SB203580 or siRNA specific to p38α MAPK. Previous studies have demonstrated that the inhibition of p38 MAPK can diminish microglial phagocytosis of injured neurons, and that phosphatidylserine (PS)-containing liposome can activate a signaling pathway inducing phagocytosis of apoptotic cells, including p38 MAPK. In addition, PS-induced activation of Rac1 was diminished in the presence of a p38 MAPK inhibitor in macrophages transfected with class B scavenger receptor type I, which is a receptor recognizing PS in testicular Sertoli cells [14, 30]. Rac1 activity was associated with augmentation of the phagocytic ability toward apoptotic cells, which was associated with the reorganization of the cytoskeleton involving microtubule and actin dynamics [25, 40].

AICAR allosterically activated AMPK and induced phosphorylation of AMPK by AMPK kinases, such as liver kinase B1, and could increase the activity of TAK1 in mammalian cells [16, 41]. TAK1, a member of the MAPK kinase kinase family, activated MAPK kinases such as MKK3/6, which phosphorylates p38 MAPK [42]. In the present experiments, inhibition of TAK1 activation using (5Z)-7-oxozeaenol diminished AICAR-induced p38 MAPK phosphorylation and phagocytosis of apoptotic cells. These results suggest that AICAR can increase p38 MAPK activity through TAK1 activation independently of AMPK activation, which could be a potential additional mechanism explaining AICAR-induced increase in efferocytosis.

Many studies have shown that clearance of external pathogens such as bacteria is important in host defense, and the effective removal of apoptotic cells improves the resolution of inflammatory conditions such as acute lung injury [4–7]. In our experiments, inhibition of TAK1 activation using (5Z)-7-oxozeaenol reduced the AICAR-induced p38 MAPK phosphorylation and phagocytosis of apoptotic cells. By demonstrating that AICAR increases the activities of p38 MAPK independently of AMPK, which participates in phagocytosis of apoptotic cells, we describe novel mechanisms for AICAR in the modulation of efferocytosis-mediated inflammatory processes and propose AICAR as a candidate pharmacological agent for the adjunctive treatment of inflammatory conditions that are clinically relevant during critical illness.
